# You Do Not Have to Get through This Alone: Interpersonal Emotion Regulation and Psychosocial Resources during the COVID-19 Pandemic across Four Countries

**DOI:** 10.3390/ijerph192315699

**Published:** 2022-11-25

**Authors:** Olenka Dworakowski, Zilla M. Huber, Tabea Meier, Ryan L. Boyd, Mike Martin, Andrea B. Horn

**Affiliations:** 1 URPP “Dynamics of Healthy Aging”, University of Zurich, 8006 Zurich, Switzerland; 2 Department of Psychology—Gerontopsychology, University of Zurich, 8050 Zurich, Switzerland; 3 School of Education and Social Policy, Northwestern University, Evanston, IL 60208, USA; 4 Independent Researcher, Washington, DC 20036, USA; 5 Competence Center Gerontology, University of Zurich, 8006 Zurich, Switzerland

**Keywords:** interpersonal emotion regulation, COVID-19, mental health, psychosocial resources

## Abstract

While experiencing the unpredictable events of the COVID-19 pandemic, we are likely to turn to people in order to regulate our emotions. In this research, we investigate how this interpersonal emotion regulation is connected to affective symptoms, above and beyond *intra*personal emotion regulation. Furthermore, we explore whether perceived psychosocial resources moderate these associations, i.e., if individuals reporting healthier social connections benefit differently from interpersonal emotion regulation. *N* = 1401 participants from the USA, UK, Germany, and Switzerland completed an online survey that included text samples. Affective symptoms (depression, adjustment disorder, fear of COVID-19) were examined based on self-reported as well as language-based indicators. As psychosocial resources, we examined social support, loneliness, attachment style, and trust. We defined latent variables for adaptive and maladaptive interpersonal emotion regulation and analyzed how they were associated with affective symptoms controlling for intrapersonal emotion regulation. Further, we analyzed how they interacted with psychosocial resources. Maladaptive interpersonal emotion regulation strategies were associated with affective symptoms. With lower psychosocial resources, the associations between interpersonal emotion regulation and depressive symptoms were more pronounced. The results highlight that maladaptive interpersonal emotion regulation is associated with worse mental health. These effects are not buffered by more psychosocial resources and are stronger for people with low psychosocial resources.

## 1. Introduction

During the COVID-19 pandemic, people were highly challenged in regulating their emotional responses to the risks resulting from the pandemic [1]. At the same time, social relationships as we know them faced a large threat caused by restrictions on social contact [2]. Some people managed to adjust to the situation and were able to use their psychosocial resources in order to stay resilient. Whereas others who perceived their psychosocial resources as limited might have profited less from those social interactions, which were still possible [3]. With this work, we want to add to the understanding of how social interactions can foster resilience by serving emotion-regulatory purposes while also investigating the role of the perceived psychosocial resources, such as social support, social inclusion, attachment style, and general trust in others, of individuals. Can healthy social relationships buffer the detrimental effects of problematic interactions while enhancing the effects of healthy interactions? 

Generally speaking, the COVID-19 pandemic posed a threat to our mental health [1]. As the pandemic is a historically unique stressful event, it is a strong risk factor for different mental health issues, as shown in an array of affective symptoms. Affective symptoms are here defined as symptoms associated with affective disorders, such as depressive, adjustment, and anxiety disorders [4,5,6,7]. Adjustment disorder is defined as a failed adjustment to a stressful event or change in one’s life [4] and, thus, a likely stress reaction to the pandemic, as shown in several studies [5]. However, depressive symptoms [1] and fear of COVID-19 [8] have also been found to be associated with problems adapting to the pandemic. Beyond self-reports, the recent literature points out the promising predictive value of features of language use as behavioral indicators of stress-related processes and negative emotionality; that is, the way we speak or express ourselves in writing can be indicative of specific affective symptoms. As an example, a high frequency of self-references, as indicated by the use of first-person singular pronouns has been found indicative of negative emotionality across diverse language and speech samples [9]. On the other hand, many people stayed resilient despite the stress of the pandemic. Emotion-regulatory processes are likely to contribute to the variability of who manages to stay resilient and who faces a harder time [10].

The ability to calibrate our emotional responses in healthy ways is at the core of adjusting to stress and life challenges and is called emotion regulation [11]. Habitually applying adaptive emotion regulation strategies is connected to healthier outcomes, whereas maladaptive strategies are associated with more detrimental outcomes [12]. Considering all strategies of emotion regulation, ruminative brooding on negative thoughts; that is, circling your thoughts around the negative content of a stressful event, has been found to be strongly connected to poorer health [13]. On the other hand, reappraising a situation, i.e., taking a new perspective on a stressful event and seeing positive aspects in it, is associated with favorable health outcomes [14]. Both strategies have been described as happening *intra*personally with oneself. During the COVID-19 pandemic, studies have already found associations between emotion regulation strategies and mental health outcomes, such as depression, adjustment disorder symptoms, and anxiety [15]. What has not been considered in many studies of emotion regulation so far, are strategies based on *inter*personal processes. According to the Social Baseline Theory [16], our regulation of emotions is, by default, embedded in our social relationships. There is increasing evidence for the importance of an interpersonal perspective in emotion regulation research since these processes often automatically happen while interacting with others [17,18,19,20]. In this paper, interpersonal emotion regulation (IER) is defined as emotion regulation in social interaction that is targeted at regulating one’s own emotions—often referred to as intrinsic as opposed to extrinsic [20,21]. This includes the social sharing of emotions and experiences, as most interpersonal emotion regulation strategies include verbal disclosure. However, this verbal disclosure or sharing can happen in more or less adaptive ways. In fact, most of the strategies found in the extensive research on intrapersonal emotion regulation (e.g., brooding and reappraisal) can happen in interaction with others [18].

Additionally to the parallel processes, as discussed in the intrapersonal emotion regulation literature, a genuinely interpersonal mechanism in IER is the idea of a socio-affective pathway of emotion regulation through changes in relationship quality [22]. This idea has been supported in studies investigating mediating processes of dyadic affective dynamics in the daily life of couples [17,23]. The notion of a socio-affective pathway has been referenced in different views on IER as the fulfillment of affiliative needs [19], social proximity [16], and affiliation, as well as safety signals [20]. Relationship science informs how the establishment of psychological intimacy lies at the core of such dyadic processes [24]. The interpersonal process model of intimacy suggests that the establishment of intimacy involves an interactive process that starts when a person discloses personal content to an interaction partner, who then reacts in a way that is perceived as responsive, which ultimately leads to a shared sense of closeness [24]. Following this framework, IER strategies work via the same pathways through which intrapersonal strategies are supposed to work [14] and additionally by altering relationship quality. Thus far, the following strategies have been found as adaptive: *co-reappraisal* (i.e., taking a new perspective with the goal of changing the emotional impact of a situation in dialogue; [22]), *co-distraction* (i.e., distracting, or being distracted from distressing situations by, for example, talking about other topics; [25]), offering *physical affection* (e.g., such as hugging; [23]), and applying *positive humor* (e.g., benevolently joking about the situation; [17]). However, mirroring processes of intrapersonal emotion regulation strategies can also be maladaptive. Ruminative *co-brooding* (i.e., rigidly circling around negative emotions in dialogue without letting one’s social partner intervene, [26]), *co-suppression* of one’s emotional expressions in social interaction, or bringing up *negative humor* (i.e., bitter irony and sarcasm), have been defined as maladaptive IER strategies [18,22]. These strategies for dealing with emotional content can be harmful in two ways; they contribute to emotional dysregulation and affective symptoms, and they may additionally deteriorate the quality of close relationships by interfering with the establishment of psychological intimacy.

From a psychopathological perspective, particularly in depression [27] and anxiety disorders [28], the interconnectedness of interpersonal processes, emotion regulation, and affective symptoms is being acknowledged more and more [22,29,30]. However, only a few studies have considered how IER acts above and beyond *intra*personal emotion regulation, which has traditionally been studied more extensively. To better understand the importance of IER, it is vital to identify its added value. Accordingly, recent studies have focused on specific interpersonal aspects of emotion regulation during the pandemic [10,31,32]. However, these studies investigated specific strategies only and did not integrate various adaptive and maladaptive IER strategies by simultaneously considering their predictive effects on affective symptoms. A better understanding of IER processes is of particular interest, as it may inform interventions on individual and societal levels bridging the seemingly conflicting findings of social sharing [19] and disclosure [24] as important adaptive interpersonal strategies when dealing with stress while other forms of sharing and disclosure—such as co-brooding—are found to be harmful to one’s own affective state and relationship quality. That is to say, it is important to better understand *what kind* of social sharing is adaptive and what other factors might influence how we regulate our emotions interpersonally.

Furthermore, it also remains unclear what role an individual’s perceived psychosocial resources play in the process of IER. As psychosocial resources, we will here focus on perceived social support, loneliness, attachment style, and general trust in others. We chose mental representations of the quality of one’s own relationships that are supposed to be important social exchange processes related to IER. The perception of being supported and socially included seems important when it comes to relying on these psychosocial resources when turning to others for regulation of one’s own emotions. Even more basic is the perceived possibility of trusting and feeling safe with others, which is reflected in a secure attachment style and reported trust in other people. Possibly, adaptive effects of specific habitual IER strategies are only able to unfold if people have enough healthy psychosocial resources available to them. In other words, if I have fewer opportunities to discuss my problems with someone, it might not make a difference if my habitual tendency to discuss them is more adaptive. On the other hand, strong psychosocial resources might buffer the effects of maladaptive IER. Feeling that we do not have any social support or feeling lonely are indicators of being isolated and were found to be predictive of psychological and physical health problems [33] as well as suicidal behavior [30] and have been connected to less positive social behavior [34]. What is more, a secure attachment style and being able to trust others are important in building social connections with others [35]. Individuals with insecure attachment styles have been shown to be at higher risk of mental health problems [36], with attachment style shown to be closely related to emotion regulation [37]. Loneliness, social support, and attachment style have all been associated with mental health during the pandemic [38,39,40,41,42]. A recent systematic review and meta-analysis have found a small but significant increase in loneliness during the pandemic [2]. Moreover, these kinds of psychosocial resources have been shown to buffer vulnerabilities resulting from maladaptive emotion regulation: The social buffer hypothesis [43] suggests that social support-related interpersonal mechanisms buffer against stress experiences and vulnerabilities. In a dyadic daily diary study, for example, responsive touch by the partner buffered against maladaptive momentary thought suppression [44]. Similarly, other studies have shown buffering effects of social support against the effects of other maladaptive emotion regulation in challenging situations [45,46].

### The Current Study

**Figure 1:**
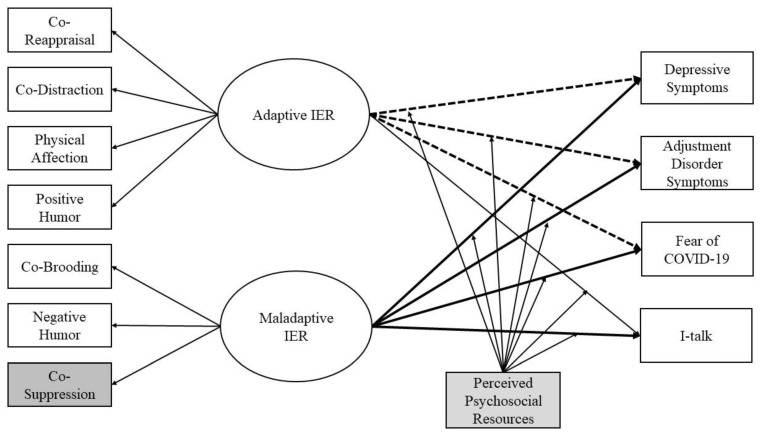
Conceptual Model. Note. IER = Interpersonal emotion regulation. The paths between IER and the affective symptom outcomes were all further controlled for age, sex, country, prior mental health diagnosis, social support, attachment style, loneliness, trust in others, and in model 1b additionally, intrapersonal emotion regulation strategies of brooding and reappraisal. Co-suppression (displayed in grey) was later excluded from the model because it did not load sufficiently on the latent variable (<0.40). The bold lines represent significant paths above and beyond intrapersonal emotion regulation, the dotted lines represent significant paths only without controlling for intrapersonal emotion regulation (see Section 3). The interactions with perceived social resources (displayed in light grey) were added in later models for each social resource (social support, attachment style, loneliness, trust) separately.

The COVID-19 pandemic presents a historically unique situation that enables us to study people in different countries facing similar threats and stressful situations. Simultaneously, the pandemic was associated with a huge variability of perceived social realities and different IER strategies. As a first goal in our study, we wanted to empirically test our theoretical assumptions (see Figure 1) of IER strategies in the context of the pandemic. We expected that these IER strategies were associated with mental health indicators during the COVID-19 pandemic, *above and beyond* intrapersonal brooding and reappraisal, as well as psychosocial resources. We further wanted to contribute to a better understanding of the interplay between IER strategies and perceived psychosocial resources. Do IER strategies have comparable effects across individuals with varying psychosocial situations? For example, if individuals feel lonely, can they still benefit from adaptive social interactions, or do they need at least some psychosocial resources for IER to be able to unfold?

To sum up, our central preregistered (https://osf.io/wqz84/?view_only=c49e39295312410f91f6220c3a31d7c6, accessed on 21 November 2022) research questions are: (1) Are more adaptive IER strategies related to better mental health outcomes during the COVID-19 pandemic? (2) Do perceived loneliness, social support, attachment style, and general trust in others moderate this relationship. We hypothesized that more adaptive interpersonal emotion regulation strategies are connected to less affective symptoms during the COVID-19 pandemic. In turn, we expected more maladaptive strategies to be associated with more affective symptoms. We expected to find associations even when controlling for intrapersonal emotion regulation strategies. Furthermore, we expected more social support, a better attachment style, less loneliness, and more trust in others to buffer the association between maladaptive IER and mental health indicators while enhancing the association with adaptive IER. Figure 1 shows the conceptual model we aimed to test in this study.

As mental health outcomes, we investigate both indicators of general mental health and affective responses specific to the pandemic. We included depressive symptoms [6], representing a more general indicator of distress. As indicators of specific stress response to the pandemic, we included adjustment disorder symptoms [4], specifically referencing the pandemic situation, as well as fear of COVID-19 [8]. In order to overcome the limitation of self-reports and following a multi-method perspective, we also included a behavioral measure, namely a language-based indicator of negative emotionality derived from text samples. Following well-established findings from the literature showing that high rates of self-reference words, what is called I-talk (i.e., I, me, my), can indicate negative emotionality and depression [9,47], as well as ruminative processing of stressful events [47], we included the rate of self-reference words in our models. Such behavioral measures are important to consider as they tap into less conscious processes that tend to be obscured by biases in self-reports [48]. High levels of I-talk are indicative of excessive self-focus, which has been connected to vulnerable narcissism [49]. Excessive self-focus is often associated with the development of depressive symptoms. In a large-scale, multi-lab, multi-method study using different types of text and speech samples, high rates of I-talk were found to be connected to negative emotionality across all different samples [9]. Such behavioral indicators are gaining more and more attention, for example, with new approaches, such as behavioral phenotyping [50].

We considered participants from four different countries in our analysis to increase the generalizability and robustness, taking advantage of the unique situation of a comparable global stressor. We will be focusing on effects across all countries instead of differences between the countries in order to investigate the underlying processes across social contexts. With the inclusion of age-diverse participants from four different countries and these diverse outcomes, as well as control variables, we aim to contribute to a clearer picture of what role IER strategies can play in a stressful situation dependent on the individual psychosocial resources at hand above and beyond intrapersonal emotion regulation strategies.

## 2. Materials and Methods

### 2.1. Participants and Procedure

Our study included a multi-national sample of adults. Participants were recruited via research participant recruitment platforms, Prolific (https://www.prolific.co/, accessed on 4 November 2020) and Respondi (https://www.respondi.com/, accessed on 4 November 2020), in the context of a larger study aiming to investigate individual and collective emotion regulation during the pandemic. Participants were eligible to participate if they were at least 18 years old and living in either the USA, UK, Switzerland, or Germany. We focused on these four countries because they are quite similar in terms of common cultural dimensions (https://www.hofstede-insights.com/, accessed on 10 June 2022). Moreover, we aimed at an age-diverse sample and thus specifically also targeted people above 65 years after having reached a sufficient number of younger participants. We used the same procedure of data collection for all countries and specifically aimed at representative samples that were equally distributed across age and gender. The descriptive statistics regarding the four samples show us comparable values in demographic variables across the four countries (see Table 1).

After informed consent, participants completed an online survey in which we also sampled participants’ written language based on common instructions to derive natural language samples for text analysis [51]. The instruction asked participants to write about their deepest thoughts and feelings regarding the COVID-19 pandemic for five to ten minutes, without thinking about grammar or spelling but just letting their thoughts run (see supplemental material SA for specific instructions). Data were collected between 4 and 17 November 2020, during the early phases of the second wave of the pandemic. At this point, there were still quite strong regulations in all countries. The mean stringency index, which describes a measure of government response to the pandemic (e.g., schools closing, cancellation of public events, etc.; as reported on https://ourworldindata.org/covid-stringency-index, accessed on 10 June 2022) for the USA was 65.74, for UK 67.99, for Germany 62.04, and for Switzerland 59.26. The situational burden was thus still relatively comparable between the four countries.

Participants were retrospectively excluded from the analysis if their data showed inconsistencies, unlikely responses, unusual comments (*N* = 37), or their text sample was under 50 words (*N* = 179), assuming that these participants did not take the task seriously. The final sample included in the analysis consisted of *N* = 1401. The mean age was 48.1 years (*SD* = 16.2; range = 18–88), and 52% were women. Table 1 shows the sample characteristics, including the participants’ sociodemographic information. The ethics committee of the university with which most authors are affiliated approved this study.

**Table 1:** Sociodemographic and Study Variables.

	USA (*N* = 383)	UK (*N* = 399)	Germany (*N* = 311)	Switzerland (*N* = 308)
	*N* (%)	*N* (%)	*N* (%)	*N* (%)
Sex: Identified Women	200 (52.10)	203 (50.90)	159 (51.10)	169 (54.90)
Living alone	61 (15.90)	42 (10.50)	80 (25.70)	70 (22.70)
Have children	178 (46.40)	154 (38.60)	183 (58.80)	171 (55.50)
In romantic relationship	270 (70.30)	300 (75.30)	206 (66.80)	226 (73.30)
College graduate (bachelor’s degree)	139 (36.20)	160 (40.10)	66 (20.20)	88 (28.50)
Vocational education and training	-	-	117 (37.6)	131 (42.5)
	M (*SD*)	M (*SD*)	M (*SD*)	M (*SD*)
Age	47.43 (17.32) Range: 18–83	47.13 (16.47) Range: 18–88	49.20 (15.56) Range: 18–82	49.13 (14.90) Range: 18–78
Depressive symptoms	7.41 (6.72)	7.05 (6.20)	5.65 (6.30)	4.90 (5.44)
Adjustment disorder symptoms	18.70 (6.28)	18.31 (5.80)	17.58 (6.17)	16.99 (5.87)
Fear of COVID-19	15.62 (6.89)	14.22 (6.25)	14.18 (6.21)	13.04 (5.63)
Self-reference	7.09 (3.20)	7.26 (2.98)	6.55 (3.78)	6.27 (4.01)

*Note. N* = count, *M* = mean, *SD* = standard deviation. Vocational education and training were only included in the German and Swiss sample. Such education is very common in these countries and would roughly correspond to an educational level of a bachelor’s degree in the USA or UK in terms of socioeconomic status. Depressive symptoms were measured with the Phq-9 [6] and has a cut-off value for clinical relevance of 10. Adjustment disorder symptoms were measured with the ADNM [4] and has a cut-off value of 19. Fear of COVID-19 was measured with the FCV-19S [8] and shows a cut-off score of 17 [52].

### 2.2. Measures

For a detailed description of all our study materials, including reliability measures, consult the supplemental material SA. Depressive symptoms were measured by the Patient Health Questionnaire [6]. Adjustment disorder symptoms were measured by the Adjustment Disorder New Module (ADNM-8; [4]). Fear of COVID-19 was measured by the Fear of COVID-19 Scale (FCV-19S; [4]). I-talk [9] was measured with the text analysis program LIWC2015 [53], and the German-adapted and validated version [54] in the text samples included in the online survey.

Interpersonal emotion regulation was measured by the Interpersonal Emotion Regulation Questionnaire IER-CR (see supplemental material for details on items and reliability [55]). Maladaptive intrapersonal strategy ruminative brooding was measured with the corresponding subscale of the Response Style Questionnaire (RSQ; [56]). As an adaptive strategy, we considered the reappraisal subscale of the Cognitive Emotion Regulation Questionnaire (CERQ; [12]) in our analysis.

Perceived social support was measured by the Brief Social Support Scale (BS6; [57]). Attachment style was measured with one item representing secure attachment [35]. Perceived loneliness was measured by a scale composed by Hughes et al. [58]. General trust in others was measured with a single item composed by the ‘More in Common’ project (https://www.moreincommon.com/newnormal/, accessed on 21 September 2020).

### 2.3. Data Analysis

We applied structural equation modeling in order to establish the association between interpersonal emotion regulation and affective symptoms during the pandemic while controlling for any covariations between the variables. All our structural equation analyses were conducted in MPlus Version 8 [59]. Following our conceptual model (Figure 1), latent variables and their association with mental health outcomes were first fitted while constraining the *intra*personal strategies paths to zero (Model 1a). In the next step, we investigated the associations between IER and affective symptom outcomes while controlling for *intra*personal strategies, thus adding reappraisal and brooding as covariates (Model 1b). We additionally calculated Model 1b while grouping the data by country in order to explore country-specific effects, rendering separate models for each country. Lastly, we investigated interaction effects between the latent variables of IER and the psychosocial resource variables in four separate models (Models 2a–d), one for each psychosocial resource we included. For a more detailed description of the data analytical procedure, consult the supplemental material SB. The research questions, measures, analyses, and hypotheses were all preregistered at: https://osf.io/wqz84/?view_only=c49e39295312410f91f6220c3a31d7c6, accessed 21 November 2022 (see: “Emotion Regulation Covid Preregistration”).

## 3. Results

Following the theoretical indication of IER, we defined two latent variables: Adaptive interpersonal emotion regulation and maladaptive interpersonal emotion regulation (see Figure 1). Our data provided support for this theoretical assumption. The adaptive IER latent variable was indicated by the following scales: Co-reappraisal (All factor loadings reported here are from Model 1b. Different models presented slightly different factor loadings, although always satisfactory (>0.50)) (0.82, *p* < 0.001, 95% *CI* = 0.79; 0.85), co-distraction (0.79, *p* < 0.001, 95% *CI* = 0.76; 0.82), physical affection (0.76, *p* < 0.001, 95% *CI* = 0.73; 0.79), and positive humor (0.77, *p* < 0.001, 95% *CI* = 0.75; 0.80). The maladaptive IER latent variable was measured by the scales co-brooding (0.87, *p* < 0.001, 95% *CI* = 0.83; 0.90) and negative humor (0.59, *p* < 0.001, 95% *CI* = 0.55; 0.63). Co-suppression was the only scale of the IER not included in the model, as it did not show sufficient loading on any of the latent variables (<0.40). The two latent variables were correlated with each other by *r* = 0.65 (*p* < 0.001, 95% *CI* = 0.60; 0.69).

Next, we calculated a structural model in which depressive symptoms, fear of COVID-19, adjustment disorder symptoms, and I-talk were associated with adaptive and maladaptive IER, as well as control variables and possible moderators (Model 1a). A summary of the stepwise results of these models can be found in Table 2. We excluded the control variables in these tables for a better overview; the results of the control variables are reported in the supplemental material SC. Here, *intra*personal emotion regulation tendencies were held at zero. In this first model, there was a significant negative association between adaptive IER and depressive symptoms (*β* = −0.54, *p* < 0.001, 95% *CI* = −0.70; −0.39), adjustment disorder symptoms (*β* = −0.26, *p* = 0.001, 95% *CI* = −0.42, −0.10), and fear of COVID-19 (*β* = −0.25, *p* = 0.003, 95% *CI* = −0.32; −0.09). Maladaptive IER, on the other hand, was associated with higher levels of depressive symptoms (*β* = 0.85, *p* < 0.001, 95% *CI* = 0.71; 0.99), adjustment disorder symptoms (*β* = 0.75, *p* < 0.001, 95% *CI* = 0.60; 0.89), and fear of COVID-19 (*β* = 0.73, *p* < 0.001, 95% *CI* = 0.59; 0.88). The model fit indices for this model were *CFI* = 0.95, *RMSEA* = 0.06, which is considered a good fit [60]. In the next step, we lifted the fixed values of the *intra*personal tendencies of brooding and reappraisal (Model 1b). Here, we no longer found any significant associations with adaptive IER. Maladaptive IER was still linked with higher levels of depressive symptoms (*β* = 0.20, *p* < 0.001, 95% *CI* = 0.10; 0.31), adjustment disorder symptoms (*β* = 0.24, *p* < 0.001, 95% *CI* = 0.12; 0.36), and fear of COVID-19 (*β* = 0.21, *p* < 0.001, 95% *CI* = 0.08; 0.35). Moreover, maladaptive IER was linked with lower levels of I-talk (*β* = −0.18, *p* = 0.021, 95% *CI* = −0.31; −0.05), which was in the opposite direction of what we had hypothesized. The model fit indices for this second model were *CFI* = 0.95, *RMSEA* = 0.06. The model comparison between the two models showed that adding reappraisal and brooding significantly improved model fit (*X*^2^ = 69.72 (8), *p* < 0.001).

We then recalculated Model 1b but by grouping the data by country. The overall model fit remained acceptable (*CFI* = 0.96; *RMSEA* = 0.05). Details of these results can be found in the supplemental material (Tables S4–S7). All in all, the results of the individual countries go in the same direction as in the overall model but are not significant for all indicators in all countries.

In the next step, we investigated the interaction effects between each attachment style, perceived social support, loneliness, as well as trust in others and the interpersonal emotion regulation latent variables (Models 2a–d). The interaction models with attachment style (*X*^2^ = 99.28 (8), *p* < 0.001) and loneliness (*X*^2^ = −2205.48 (8), *p* < 0.001) showed significant *X*^2^ values in the difference test, indicating better model fit compared to the models without interaction effects. The models for interactions with perceived social support (*X*^2^ = 6.76 (8), *p* = 0.56) and trust (*X*^2^ = 11.85 (8), *p* = 0.16) did not show significant *X*^2^ in the difference test, indicating no significant improvement in model fit compared to the model without interaction effects. The results of all interaction models are summarized in Table 3. We excluded the control variables in these tables for a better overview; the results of the control variables are reported in the supplemental material SC.

**Figure 2:**
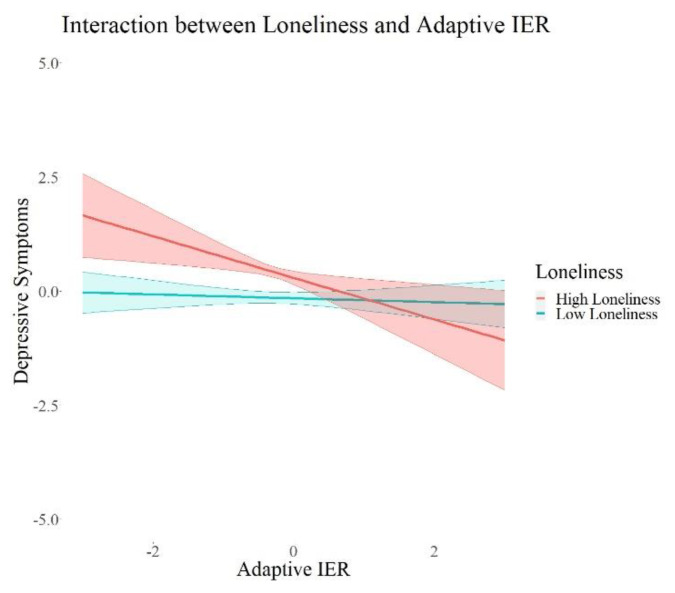
Interaction Effects between Loneliness and Adaptive IER on Depressive Symptoms. Note. The shades behind the thicker lines represent the 95% confidence interval. All variables were standardized, and the scales on the graph represent standard deviations (i.e., 2 = 2 units of standard deviation).

**Figure 3:**
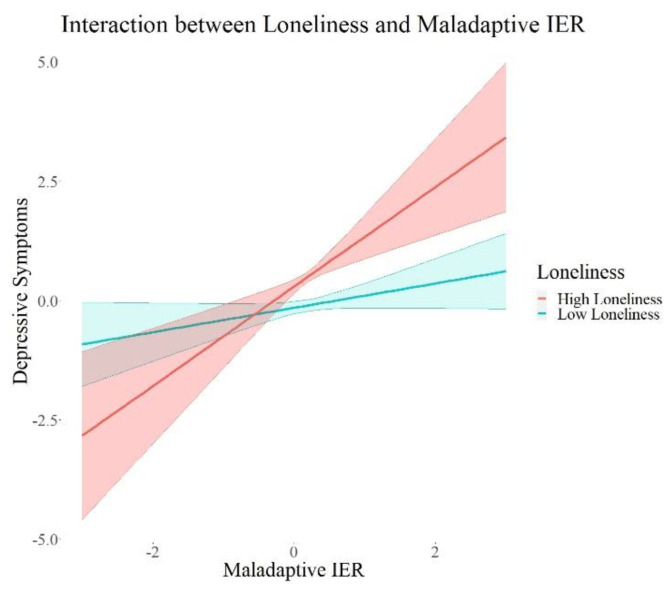
Interaction Effects between Loneliness and Maladaptive IER on Depressive Symptoms. Note. The shades behind the thicker lines represent the 95% confidence interval. All variables were standardized, and the scales on the graph represent standard deviations (i.e., 2 = 2 units of standard deviation).

**Figure 4:**
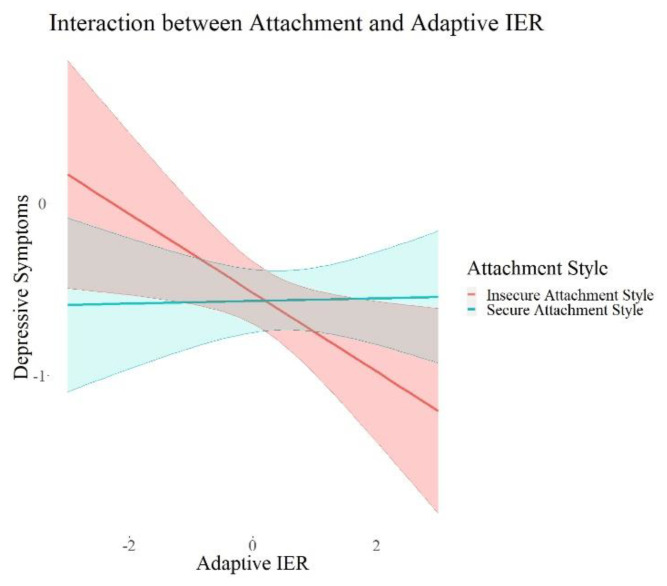
Interaction Effects between Attachment and Adaptive IER on Depressive Symptoms. Note. The shades behind the thicker lines represent the 95% confidence interval. All variables were standardized, and the scales on the graph represent standard deviations (i.e., 2 = 2 units of standard deviation).

**Figure 5:**
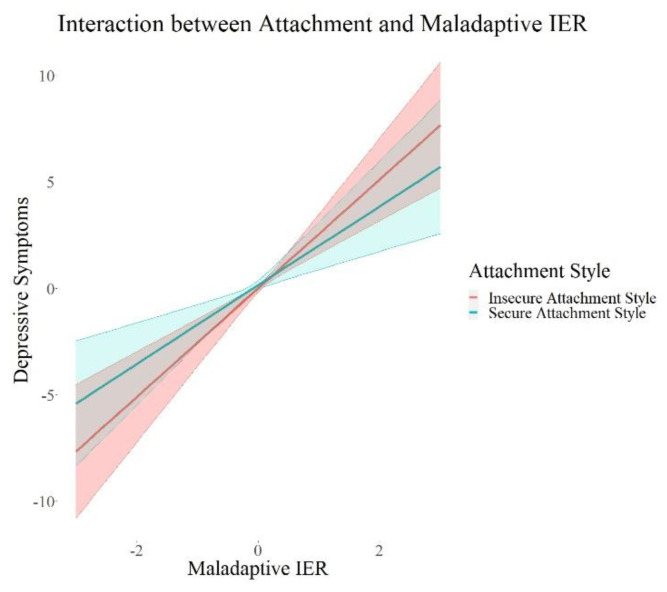
Interaction Effects between Attachment and Maladaptive IER on Depressive Symptoms. Note. The shades behind the thicker lines represent the 95% confidence interval. All variables were standardized, and the scales on the graph represent standard deviations (i.e., 2 = 2 units of standard deviation).

**Figure 6:**
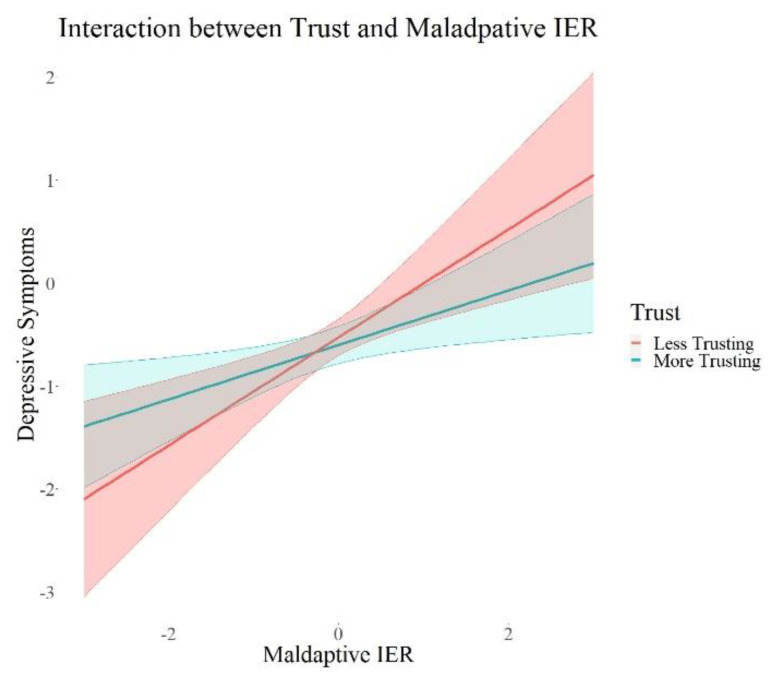
Interaction Effects between Trust and Maladaptive IER on Depressive Symptoms. *Note.* The shades behind the thicker lines represent the 95% confidence interval. All variables were standardized, and the scales on the graph represent standard deviations (i.e., 2 = 2 units of standard deviation).

We found a significant positive interaction between attachment style (*β* = 0.09, *p* = 0.015, 95% *CI* = 0.02; 0.17) and adaptive interpersonal emotion regulation, as well as a negative interaction of loneliness (*β* = 0.17, *p* = 0.001, 95% *CI* = −0.27; −0.07) and adaptive interpersonal emotion regulation on depressive symptoms. Further, we found significant negative interactions between each attachment style (*β* = −0.10, *p* = 0.02, 95% *CI* = −0.18; −0.02) and trust (*β* = −0.08, *p* = 0.05, 95% *CI* = −0.15; −0.00) and maladaptive interpersonal emotion regulation, as well as a significant positive interaction between loneliness (*β* = 0.23, *p* < 0.001, 95% *CI* = 0.13; 0.34) and maladaptive interpersonal emotion regulation on depressive symptoms. There were no interaction effects on adjustment disorder symptoms, fear of COVID-19, or I-talk. Maladaptive IER remained robust in its positive association with depressive symptoms and adjustment disorder symptoms in all these models. I-talk remained significantly negatively associated with maladaptive interpersonal emotion regulation in the models with interactions with social support (Model 2a) and loneliness (Model 2c). Maladaptive IER was still significantly positively associated with fear of COVID-19 in all models except for the one with interactions with attachment style (Model 2b). In summary, we found interaction effects between each attachment, loneliness, as well as trust and maladaptive IER, and an interaction between loneliness and adaptive IER. All the interactions were only associated with depressive symptoms. In order to visualize this effect, we plotted the associations of adaptive and maladaptive IER with depressive symptoms for each group of high and low psychosocial resources in Figure 2, Figure 3, Figure 4, Figure 5 and Figure 6. These show how the slopes for people with low psychosocial resources are steeper and how the effects are significant only for people with weaker tendencies to adaptive or stronger tendencies to maladaptive IER strategies. The plots of attachment style and trust show overlapping confidence intervals of the two groups. Overlapping confidence intervals of the two groups do not indicate that this effect is not significant [61]. The probabilities of finding non-overlapping confidence intervals between two groups are very small (0.0056 if the variance of effect estimates are equal and independent, see [62] for details of this calculation) and often mistakenly interpreted as the only sign of significance [62]. Table 3 shows that the interaction effects displayed on the plots are significant, defined by a *p*-value > 0.05 and a confidence interval not containing zero. The interaction effects with attachment style and trust show smaller effect sizes than the interactions with loneliness, which is why we might see these overlapping confidence intervals of the two groups here.

## 4. Discussion

The goal of this study was to investigate the role of adaptive and maladaptive IER strategies on affective symptoms across four countries during the beginning of the COVID-19 pandemic. We aimed to adequately conceptualize IER strategies and investigate whether individual psychosocial resources make a difference in how IER and mental health are related. We used the COVID-19 pandemic as an opportunity to examine the same collective stressor across different participants and countries. We believe that the research questions we address in this study are relevant beyond the situation of the COVID-19 pandemic. The pandemic proposes a unique situation in which we can study participants facing the same real-life stressor. Nonetheless, emotion regulation is important for mental health across different stressful situations one might encounter, and thus, our results are likely to be generalized beyond the pandemic, and further research is warranted. In line with our hypotheses, the results showed a significant effect of maladaptive IER strategies on mental health indicators, above and beyond intrapersonal strategies and perceived psychosocial resources. Furthermore, we found interaction effects between adaptive as well as maladaptive IER strategies and psychosocial resources on depressive symptoms.

In partial support of our first hypothesis, our results highlight the important role IER strategies can play in mental health above and beyond intrapersonal strategies. Our results show that affective symptoms were associated with more maladaptive IER strategies, as indicated by higher levels of co-brooding and negative humor. In other words, individuals who reported engaging in repetitive, rigid exchanges about negative content with their close ones and more use of bitter sarcasm reported more depressive symptoms, issues in adjusting to the pandemic, and more fear of COVID-19. This association stayed robust when controlling for intrapersonal brooding and reappraisal. This suggests that even if these interpersonal strategies would be seen as interpersonal manifestations of individual emotion regulation habits, performing them in dialogue has an additional impact on mental health outcomes. This supports the notion of a related but independent socio-affective pathway of IER in explaining mental health symptoms, in addition to intrapersonal emotion regulation.

Adaptive IER strategies showed the expected negative associations with affective symptoms, suggesting a protective function on the adjustment to the pandemic. People who indicated often trying to take a new perspective on things in exchange with their close ones, engaging in positive humor [17], and relying on the exchange of physical affection [23] reported fewer symptoms of depression, better adjustment to the pandemic, and less fear of COVID-19. However, the effects were smaller than for the maladaptive pattern of IER and did not hold when controlling for intrapersonal emotion regulation strategies. This suggests that adaptive IER strategies did not protect individuals from distress beyond intrapersonal strategies of emotion regulation in our sample. Mirroring results have been found in research on intrapersonal strategies [12], where maladaptive individual emotion regulation tendencies showed a stronger, more robust association with mental health issues. In our analysis, intrapersonal brooding was also significantly associated with depressive symptoms, as well as adjustment disorder symptoms and fear of COVID-19, while intrapersonal reappraisal was only associated with depressive symptoms. Such somewhat inconsistent results for reappraisal have also been found in previous studies [15]. This indicates that, generally, maladaptive strategies seem to be more harmful than adaptive strategies and are protective in both intra- and interpersonal forms of emotion regulation.

During the pandemic, social factors played a prominent role [32]. Multiple studies—including ours—report results showing how social sharing may not always be adaptive [10] but might be associated with negative effects on mental health and relationship quality if sharing is rigid and negative, as is co-brooding, for example. On the other hand, studies on post-traumatic growth have shown the positive potential of deliberately sharing negative content [22] as opposed to rigidly and repetitively sharing negative content without being open to new perspectives. It is important to point out that social sharing and self-disclosure are important in times of stress [29], but it also matters how people do it.

Our study also included a behavioral measure of negative emotionality, I-talk [9]. Results concerning I-talk were somewhat different from those of the other indicators of affective symptoms. More specifically, maladaptive IER strategies were associated with less I-talk. This may seem contradictory at first, as previous research has found more I-talk to be indicative of negative emotionality and depression [9]. Recently, Berry-Blunt et al. [49] discussed that I-talk might reflect a self-focus that is related to a form of narcissistic focus, implying vulnerability. This view may explain why I-talk was positively connected to negative emotionality in our study but not to maladaptive IER. However, there was a positive (but non-significant) relationship between I-talk and adaptive interpersonal emotion regulation. These heterogeneous results are in line with earlier discussions of how language indicators are always determined by a multitude of factors, and self-focus does not always have to be negative [49]. It might be that a tendency to adaptive IER is associated with healthier self-reflection [10]—possibly including functional expressions of vulnerability and explicit labeling of emotions [20]. It is important to note that we only have written language samples within the online assessment. Oral language or language in other contexts might show different results, even though I-talk has been shown to be quite a robust indicator across contexts [9].

Concerning our second research question, we found significant interaction effects between both adaptive and maladaptive IER strategies and psychosocial resources, while the IER main effects remained significant, as expected. In contrast to our expectations, psychosocial resources only moderated the effects of IER on depressive symptoms and not on stress-related symptoms. In other words, the effects of how I speak about what is distressing to me with others on adjustment problems or anxieties, specifically about the COVID-19 pandemic, remains the same no matter if I feel more or less supported by, attached to, connected to, and trusting of the people around me. Previous studies have also found differential effects between stress response-related and more general mental health indicators during the pandemic [3]. Our interaction results are in line with interpersonal theories of depression [30]. These suggest interpersonal issues be an etiological factor contributing to the development of depressive symptoms [27].

Furthermore, against our expectation formulated in our second hypothesis, more psychosocial resources did not buffer the effect of maladaptive IER strategies. Interaction effects indicate how people who might have less psychosocial resources, i.e., have less perceived social support, show an insecure attachment style, are lonelier, and have difficulty trusting others, benefit more from adaptive while carrying more harm from maladaptive interpersonal emotion regulation strategies. In other words, it seems as though IER strategies, in general, make a larger difference when psychosocial resources are low. This idea is in line with the notion that interpersonal and environmental etiological factors of depression contribute to stress generation, ending up in a vicious cycle resulting in more and more maladaptive social environments [63]. For therapists and other practitioners, this could imply a focus on building up adaptive IER strategies when patients present low psychosocial resources. It would have been possible that the adaptive effects of IER are more beneficial when a certain level of psychosocial resources is given, as we had hypothesized beforehand. Our results do not support this notion but rather show how healthy IER strategies are even more important and impactful when individuals perceive themselves as lonely, insecurely attached, and not very trusting. This is also in line with the conceptual assumption that adaptive IER strategies involve processes that are helpful for building healthy relationships and enhancing relationship quality.

Next to the emotion regulation strategies, we added perceived psychosocial resources as predictive variables of affective symptoms in our models. It is worthwhile to note that in our models social support, attachment style, and trust did not show any association with affective symptoms beyond emotion regulation strategies. This is contradictory to previous findings [39,40]. According to our results, these psychosocial resources are less impactful for affective symptoms than strategies of emotion regulation. Loneliness did show significant associations with all our mental health indicators and thus seems to be the strongest psychosocial risk factor. Several studies, including a meta-analysis, have shown increases in loneliness since the beginning of the pandemic [2,41,64,65,66,67]. Although our study cannot give any insight into changes in levels of loneliness, it further highlights how feelings of being isolated are high-risk for our mental health. Ernst et al. [2] highlighted specific risk factors for developing loneliness; our study describes further risks when one already feels lonely: maladaptive IER strategies can multiply the adverse effects of loneliness on mental health. It is important for us to take measures against loneliness when restrictions force us to isolate and distance ourselves physically from others. For example, we can focus on and invest in our close relationships. As our results also indicate and previous authors have stated: we do not need a large social network, but rather a healthy one in order to not feel alone [68].

### Limitations

Our data are cross-sectional, and thus, all our results are merely correlational. Recent research has discussed that maladaptive emotion regulation might be a symptom as well as the cause of mental health issues [69]. However, in the context of rumination research, a bi-directional association of causing as well as maintaining and aggravating symptoms has been discussed [70]—a framework that would also be plausible to apply with IER. Our study cannot contribute to any statements of causality. Still, we show associations between IER, mental health, and other social resources that have not been connected before while controlling for previous mental health diagnoses. Even though we included diverse outcome measures, it is also difficult to interpret if these results are specific to the pandemic situation or if they are generalizable. Future studies should try to replicate our findings in other situations.

A further limitation we must recognize Is that even though we collected a sample of four different countries, they are all WEIRD (e.g., western, educated, industrialized, rich, and democratic) countries. This limits the generalizability of our results. Nonetheless, we believe that our large and evenly distributed sample size, with comparable demographics across the four countries, gives our study strength. Our results hold when controlling for country, as we did in our models. The investigation of specific country differences was beyond the scope of our paper. However, it is worthwhile to note that group comparisons revealed subtle differences between countries regarding the patterns of associations between IER strategies and different outcome variables. This is in line with research that has found that individual emotion regulation strategies show different associations with mental health during the COVID-19 pandemic between countries [71].

## 5. Conclusions

Across our lifespan, we face different types of challenges that shape us. The pandemic has been a historic normative challenge that basically everyone around the world had to face at the same time [72]. It is important for us to better understand what psychological impacts the pandemic has had and still has. During times of social distancing, when the risk of feeling isolated is high [2] and all our psychosocial resources are being challenged, it is even more important to foster healthy IER strategies since interpersonal contact—and thus opportunities to co-regulate—are limited.

Our study adds to the current literature in that it highlights the important role interpersonal emotion regulation strategies play on our mental health above and beyond intrapersonal strategies. Our results indicate that especially maladaptive, interpersonal emotion regulation could be a risk factor for mental health issues. Even more so for people who have low social resources, fostering healthy interpersonal emotion regulation strategies could thus be very important. Previous authors have highlighted the importance of emotion regulation as a target of interventions during times of pandemic [3]; we would additionally advise targeting interpersonal strategies.

## Figures and Tables

**Table 2:** Results of structural models.

	Model 1a				Model 1b			
	Depressive symptoms	Adjustment disorder symptoms	Fear of COVID-19	Self-reference	Depressive symptoms	Adjustment disorder symptoms	Fear of COVID-19	Self-reference
Adaptive IER	−0.54 *** [−0.70; −0.39]	−0.26 *** [−0.42, −0.10]	−0.25 *** [−0.32; −0.09]	0.05 [−0.08; 0.18]	−0.06 [−0.16; 0.03]	0.08 [−0.03; 0.19]	0.09 [−0.03; 0.21]	0.11 [−0.01; 0.24]
Maladaptive IER	0.85 *** [0.71; 0.99]	0.75 *** [0.60; 0.89]	0.73 *** [0.59; 0.88]	−0.11 [−0.24; 0.03]	0.20 *** [0.10; 0.31]	0.24 *** [0.12; 0.36]	0.21 ** [0.08; 0.35]	−0.18 ** [−0.31; −0.05]
Brooding	0.00	0.00	0.00	0.00	0.28 *** [0.23; 0.34]	0.25 *** [0.18; 0.32]	0.27 *** [0.19; 0.35]	0.03 [−0.04; 0.11]
Reappraisal	0.00	0.00	0.00	0.00	−0.10 *** [−0.14; −0.05]	−0.01 [−0.07; 0.04]	−0.00 [−0.06; 0.05]	−0.06 [−0.11; 0.01]
Social Support	0.04 [−0.03; 0.10]	0.03 [−0.04; 0.10]	0.01 [−0.06; 0.08]	−0.01 [−0.08; 0.05]	−0.02 [−0.06; 0.03]	−0.01 [−0.07; 0.05]	−0.03 [−0.09; 0.03]	−0.02 [−0.07; 0.04]
Attachment	0.05 ^†^ [−0.01; 0.11]	0.06 [−0.00; 0.12]	0.07 * [0.00; 0.13]	−0.01 [−0.07; 0.05]	−0.02 [−0.07; 0.02]	0.01 [−0.05; 0.06]	0.01 [−0.04; 0.07]	−0.02 [−0.08; 0.04]
Loneliness	0.20 *** [0.12; 0.27]	0.15 *** [0.08; 0.23]	0.05 [−0.03; 0.13]	0.10 ** [0.02; 0.17]	0.27 *** [0.21; 0.32]	0.20 *** [0.14; 0.26]	0.09 ** [0.03; 0.16]	0.10 ** [0.03; 0.16]
Trust	−0.02 [−0.07; 0.03]	−0.02 [−0.08; 0.03]	−0.03 [−0.09; 0.03]	−0.02 [−0.07; 0.04]	−0.04 [−0.08; 0.00]	−0.03 [−0.08; 0.02]	−0.04 [−0.07; 0.01]	−0.02 [−0.07; 0.04]
CFI	0.95				0.95			
RMSEA	0.06				0.06			
*X* ^2^	500.07 (89) ***				431.73 (81) ***			
*X*^2^ difference					69.72 (8) ***			

Note. All dependent variables were further controlled for sex, age, previous mental health diagnosis, and country (dummy codes for UK, Germany, and Switzerland). Significance codes *** *p* < 0.001; ** *p* < 0.01; * *p* < 0.05, ^†^ *p* > 0.05, and a 95% *CI* that does not contain 0. In [] is a 95% *CI*. IER stands for interpersonal emotion regulation. Brooding and reappraisal were held at 0 in Model 1a. IER was measured by the IER-CR [55] Adaptive and Maladaptive IER were calculated as latent variables (see main text for more information). Brooding was measured by the RSQ [56] and reappraisal by the CERQ [12].

**Table 3:** Interaction Models.

**Model 2a: Interaction Social Support**					**Model 2b: Interaction Attachment**				
	Depressive symptoms	Adjustment disorder symptoms	Fear of COVID-19	Self-reference		Depressive symptoms	Adjustment disorder symptoms	Fear of COVID-19	Self-reference
Adaptive IER	−0.08 [−0.19; 0.03]	0.07 [−0.05; 0.19]	0.08 [−0.05; 0.21]	0.13 [0.01; 0.27]	Adaptive IER	−0.18 * [−0.33; −0.02]	0.09 [−0.07; 0.24]	0.09 [−0.08; 0.26]	0.07 [−0.10; 0.23]
Maladaptive IER	0.23 *** [0.09; 0.35]	0.25 *** [0.11; 0.39]	0.22 ** [0.07; 0.38]	−0.21 * [−0.36; −0.05]	Maladaptive IER	0.34 *** [0.16; 0.52]	0.23 * [0.05; 0.41]	0.17 [0.02; 0.36]	−0.10 [−0.29; 0.08]
Support X Adaptive	0.06 [−0.00; 0.13]	0.00 [−0.07; 0.07]	−0.02 [−0.11; 0.07]	−0.02 [−0.09; 0.06]	Attachment X Adaptive	0.09 * [0.02; 0.17]	−0.01 [−0.09; 0.07]	−0.01 [−0.09; 0.08]	0.05 [−0.04; 0.14]
Support X Maladaptive	−0.04 [−0.11; 0.03]	0.02 [−0.05; 0.10]	0.02 [−0.07; 0.11]	0.03 [−0.05; 0.11]	Attachment X Maladaptive	−0.10 * [−0.18; −0.02]	0.02 [−0.05; 0.10]	0.05 [−0.04; 0.14]	−0.08 [−0.17; 0.01]
Brooding	0.28 *** [0.21; 0.34]	0.25 *** [0.17; 0.32]	0.27 *** [0.18; 0.35]	0.04 [−0.04; 0.12]	Brooding	0.27 *** [0.20; 0.34]	0.25 *** [0.17; 0.32]	0.27 *** [0.18; 0.35]	0.03 [−0.05; 0.11]
Reappraisal	−0.10 *** [−0.14; −0.05]	−0.01 [−0.07; 0.04]	−0.00 [−0.06; 0.06]	−0.06 [−0.12; −0.01]	Reappraisal	−0.09 *** [−0.14; −0.05]	−0.01 [−0.07; 0.04]	−0.00 [−0.06; 0.05]	−0.05 [−0.11; 0.01]
Social Support	−0.01 [−0.06; 0.05]	−0.01 [−0.07; 0.05]	−0.03 [−0.10; 0.04]	−0.02 [−0.09; 0.05]	Social Support	−0.02 [−0.06; 0.03]	−0.01 [−0.07; 0.05]	−0.03 [−0.09; 0.03]	−0.02 [−0.09; 0.04]
Attachment	−0.02 [−0.07; 0.02]	0.01 [−0.05; 0.06]	0.01 [−0.04; 0.07]	−0.02 [−0.08; 0.04]	Attachment	−0.02 [−0.07; 0.02]	0.01 [−0.05; 0.06]	0.01 [−0.04; 0.07]	−0.02 [−0.08; 0.04]
Loneliness	0.26 *** [0.20; 0.32]	0.20 *** [0.14; 0.27]	0.09 ** [0.03; 0.16]	0.10 ** [0.03; 0.17]	Loneliness	0.25 *** [0.19; 0.31]	0.20 *** [0.14; 0.26]	0.10 ** [0.03; 0.16]	0.09 ** [0.02; 0.16]
Trust	−0.03 [−0.08; 0.01]	−0.03 [−0.08; 0.02]	−0.04 [−0.09; 0.01]	−0.02 [−0.07; 0.04]	Trust	−0.04 [−0.08; 0.00]	−0.03 [−0.08; 0.02]	−0.04 [−0.09; 0.01]	−0.02 [−0.07; 0.04]
**Model 2c: Interaction Loneliness**					**Model 2d: Interaction Trust**				
	Depressive symptoms	Adjustment disorder symptoms	Fear of COVID-19	Self-reference		Depressive symptoms	Adjustment disorder symptoms	Fear of COVID-19	Self-reference
Adaptive IER	−0.20 * [−0.39; −0.02]	0.02 [−0.13; 0.17]	0.07 [−0.10; 0.23]	0.15 [−0.01; 0.31]	Adaptive IER	−0.15 [−0.31; 0.01]	0.05 [−0.11; 0.21]	0.04 [−0.13; 0.21]	0.12 [−0.04; 0.29]
Maladaptive IER	0.39 *** [0.16; 0.62]	0.32 *** [0.14; 0.50]	0.24 * [0.04; 0.44]	−0.23 * [−0.41; −0.05]	Maladaptive IER	0.31 *** [0.12; 0.50]	0.24 ** [0.06; 0.42]	0.23 * [0.03; 0.44]	−0.16 [−0.34; −0.02]
Loneliness × Adaptive	−0.17 *** [−0.27; −0.07]	0.02 [−0.06; 0.11]	0.09 [−0.02; 0.19]	0.04 [−0.04; 0.11]	Trust × Adaptive	0.07 [−0.00; 0.13]	0.02 [−0.05; 0.09]	0.04 [−0.04; 0.12]	−0.01 [−0.08; 0.07]
Loneliness × Maladaptive	0.23 *** [0.13; 0.34]	−0.06 [−0.14; 0.03]	−0.07 [−0.18; 0.04]	0.01 [−0.08; 0.09]	Trust × Maladaptive	−0.08 * [−0.15; −0.00]	0.02 [−0.06; 0.09]	−0.00 [−0.08; 0.08]	−0.03 [−0.11; 0.05]
Brooding	0.23 *** [0.14; 0.32]	0.23 *** [0.14; 0.31]	0.26 *** [0.17; 0.35]	0.05 [−0.04; 0.13]	Brooding	0.27 *** [0.20; 0.34]	0.24 *** [0.17; 0.32]	0.26 *** [0.18; 0.35]	0.04 [−0.04; 0.12]
Reappraisal	−0.06 * [−0.12; −0.01]	−0.00 [−0.06; 0.05]	−0.00 [−0.06; 0.06]	−0.06 [−0.12; −0.01]	Reappraisal	−0.09 *** [−0.14; −0.05]	−0.01 [−0.07; 0.04]	0.00 [−0.06; 0.06]	−0.05 [−0.12; 0.01]
Social Support	−0.02 [−0.06; 0.04]	−0.01 [−0.07; 0.05]	−0.03 [−0.09; 0.03]	−0.02 [−0.09; 0.05]	Social Support	−0.01 [−0.06; 0.04]	−0.01 [−0.07; 0.05]	−0.03 [−0.09; 0.04]	−0.02 [−0.09; 0.04]
Attachment	−0.00 [−0.05; 0.05]	0.01 [−0.04; 0.07]	0.01 [−0.04; 0.07]	0.02 [−0.09; 0.05]	Attachment	−0.02 [−0.07; 0.02]	0.01 [−0.05; 0.06]	0.01 [−0.05; 0.07]	−0.02 [−0.08; 0.04]
Loneliness	0.19 *** [0.12; 0.26]	0.20 *** [0.13; 0.26]	0.11 ** [0.04; 0.18]	0.11 ** [0.04; 0.18]	Loneliness	0.26 *** [0.20; 0.32]	0.20 *** [0.13; 0.26]	0.09 ** [0.02; 0.15]	0.10 ** [0.03; 0.17]
Trust	−0.03 [−0.07; 0.01]	−0.03 [−0.07; 0.02]	−0.04 [−0.09; 0.01]	−0.02 [−0.08; 0.04]	Trust	−0.04 [−0.08; 0.00]	−0.03 [−0.08; 0.02]	−0.04 [−0.09; 0.01]	−0.02 [−0.07; 0.04]

Note. All dependent variables were further controlled for sex, age, previous mental health diagnosis, and country (dummy codes for UK, Germany, and Switzerland). Significance codes *** *p* < 0.001; ** *p* < 0.01; * *p* < 0.05, and a 95% *CI* that does not contain 0. In [] is a 95% *CI*. IER stands for interpersonal emotion regulation. Brooding and reappraisal were held at 0 in Model 1a.

## Data Availability

The data presented in this study are available on request from the corresponding author.

## Supplementary Materials

The following supporting information can be downloaded at: https://www.mdpi.com/article/10.3390/ijerph192315699/s1. Refs. [4,6,8,9,10,12,13,35,53,54,55,56,57,58,59,73,74,75,76,77,78] are cited in Supplementary Materials file.
