# Supplementary material for: You Do Not Have to Get through This Alone: Interpersonal Emotion Regulation and Psychosocial Resources during the COVID-19 Pandemic across Four Countries
Source: Int J Environ Res Public Health. 2022 Nov 25;19(23):15699. doi: 10.3390/ijerph192315699 (PMC9735908; doi:10.3390/ijerph192315699)
Supplement: Supplementary file 1 [file ijerph-19-15699-s001.zip › ijerph-1992896-supplementary.pdf]

Supplemental Material

You do not have to get through this alone: Interpersonal emotion regulation and psychosocial resources during the COVID-19 pandemic across four countries

Contents:

SA: Materials 2

SB: Data Analysis 4

SC: Tables

Table S1: IER Items 7

Table S2: Results of model 1a & b for control variables 8

Table S3: Results of model 2a – d for control variables 9

Table S4: Results of structural model for USA 10

Table S5: Results of structural model for UK 10

Table S6: Results of structural model for Germany 11

Table S7: Results of structural model for Switzerland 11

SA: Materials

Indicators of affective symptoms

As mental health indicators of affective stress-response symptoms, we examined *depressive symptoms, adjustment disorder symptoms, fear of Covid-19,* and *I-talk*. Depressive symptoms were measured with the *PHQ9* [6], which consist of nine items, each measured on a four point Likert-type scale. This scale showed good reliability (α = .92). Adjustment disorder symptoms were measured by the Adjustment Disorder New Module (*ADNM-8*; [4]), which uses eight items with a four-point Likert-type scale. The ADNM-8 follows the stress-response perspective of adjustment disorder proposed by the ICD11 [73]. Two main symptom groups are measured, preoccupation and failure to adapt. The items can be tailored to a specific stressor, which in our study was predefined as the current pandemic situation. An example item for preoccupation is: ”I have to think about the corona-pandemic a lot and this is a great burden to me”, and for failure to adapt: “Since the corona-pandemic, I can no longer sleep properly.” The ADNM-8 global score showed good reliability (α = .91). Fear of COVID-19 was measured by the Fear of COVID-19 Scale (*FCV-19S;* [8]). This scale measures the extent of fears, worries, and anxieties around COVID-19. An example item is: “My heart races or palpitates when I think about getting coronavirus-19.” This scale was only available in English language. In order to apply the scale to our German speaking sample the scale was translated by the senior author and re-translated by the first author who is native in both languages. The scale consists of seven items on a five point Likert-type scale and showed high internal consistency (α = .91).

Lastly, *I-talk* [9] was measured with the text analysis program LIWC2015 [53] and the German adapted and validated version [54] in the text samples included in the online survey. The specific instructions for the writing task was as follows:

> “In the next five to ten minutes, we ask you to write down your deepest thoughts and emotions about your personal experience during the COVID-19-Pandemic. The pandemic has brought many social changes all over the word. People in most societies were very limited and restricted in their social activities and some of these restrictions last until today.
> For many people the current situation is a source of stress and can have a strong influence on their mood and emotional experience. How are you and your social circle doing? What did you do to find a good way to cope with the situation? How did the people around you react? What was especially difficult during this time? Did this experience have an influence on how you see yourself as a person or how you want to live your life? Or what is important to you in life? How do you connect this experience to your past, your current situation and your future? Or how you were before, how you are now and what you want to be like?
> Really let go and explore the experience of this pandemic and how it has affected you. Let your thoughts and emotions run free. Please write continuously without interruption. Do not worry about grammar, spelling, punctuation or style. Just start writing.”

LIWC2015 counts words and categorizes them into psychologically meaningful categories (e.g., “social processes”, or “first-person singular pronouns”). A category is then represented as the percentage of words belonging to that category in relation to the entire text.

Interpersonal emotion regulation

Interpersonal emotion regulation was measured by the Interpersonal Emotion Regulation Questionnaire *IER-CR* [55]. The *IER-CR* consists of seven subscales, each measured by two items, except for co-brooding which is measured by three. *Co-distraction* (α = .71), *co-reappraisal* (α = .89), *positive humor* (α = .91), and *physical affection* (α = .87) are categorized as adaptive interpersonal emotion regulation strategies. *Negative humor* (α = .83), co-brooding (α = .80), and *co-suppression* (α = .70), in turn, are seen as maladaptive strategies. All items are measured on a five point Likert-type scale and showed good reliability. The full questionnaire can be found in Table S1 below.

Intrapersonal emotion regulation

As intrapersonal emotion regulation we included established measures of adaptive and maladaptive intrapersonal strategies based on meta-analytical evidence and earlier studies [12;13]. As maladaptive strategy *ruminative brooding* was measured with the corresponding subscale of the Response Style Questionnaire (*RSQ*; [13]). This subscale consists of five items with a five-point Likert-type scale and showed high internal consistency (α = .88). Further, as adaptive strategy we

considered the *reappraisal* subscale of the *Cognitive Emotion Regulation Questionnaire* (*CERQ* [13]) in our analysis. This subscale consists of two items on a five point Likert-type scale and showed high reliability (α = .77).

Psychosocial resources

As psychosocial resources we considered perceived social support, attachment style, loneliness, and general trust in others. *Perceived social support* was measured by the *Brief Social Support Scale* (*BS6* [57]), which measures emotional and tangible support in six items across a four-point Likert-type scale. This measure showed high internal consistency (α = .92). *Attachment style* was measured with four items each representing a different attachment style [35], only one of which is considered *secure attachment*. We included a dummy code in our analysis with 1 for participants who indicated secure attachment style, and 0 for participants who indicated any other attachment style. *Perceived loneliness* was measured by three items on a three point Likert-type scale composed by Hughes et al. [58]. This scale showed high reliability (α = .81). *General trust in others* was measured with a single item where participants could choose one of two statements 'most people are trustworthy' and 'one can never be careful enough when dealing with other people'. This item was taken over from the 'More in Common' project (https://www.moreincommon.com/newnormal/).

SB: Data Analysis

A retrospective analysis of minimal detectable slope for a simple regression between standardized variables with *SD* = 1 for our sample size with a power of 80% and significance level of .05 rendered a minimal detectable effect of β = .07, suggesting the sample size was appropriate to detect the assumed effects

We applied structural equation modeling in order to establish the association between interpersonal emotion regulation and affective symptoms during the pandemic while controlling for any covariations between the variables. All our structural equation analyses were conducted in MPlus Version 8 [59]. Following our preregistered procedure, we first established the latent variables of adaptive and maladaptive interpersonal emotion regulation. We applied item parceling and used the established subscales as indicators for the latent variables. Item parceling is seen legitimate given the goal of the study was not to establish an exact factor structure, but rather to test the structural model, examining the associations between IER and affective symptoms [74]. Following recommendations [75], indicators were excluded if they loaded less than .40 on the latent variables. First, we added the paths of the latent variables and control variables to the model predicting the affective symptom outcomes, while holding the *intra*personal strategies at zero (Model 1a). In the next step, we investigated the associations between IER and affective symptom outcomes while controlling for *intra*personal strategies, thus adding reappraisal and brooding as covariates (Model 1b).

Lastly, we investigated interaction effects between the latent variables of IER and the psychosocial resource variables in four separate models (Models 2a-d), one for each psychosocial resource we included. A model with multiple latent interaction terms could not be computed as the X^2-value rendered negative. Interactions with latent variables must be computed in a random type analysis with montecarlo integration in the Mplus Version 8. This procedure does not directly calculate X^2-values and thus do not allow for automated model comparisons, as was conducted in the previous steps. For each model we calculated the X^2 for comparison with the model without the interaction effects using the log-likelihood information [76]. Exact descriptions of calculations and procedure of these tests are provided below.

We included age, sex, country of residence (as dummy codes with the USA as reference category), and previous diagnoses of psychological disorders as control variables. The item to previous psychological disorders was added as an indicator for mental health risk pre-COVID-19. This is typically done in epidemiological studies [77]. Multi-level analysis is not recommended for only four countries, as these do not represent enough nesting units [78]. Accordingly, the ICC of multilevel models with country as random intercept rendered very low (<.18), further speaking against a multi-level analysis strategy. In order to consider the nested structure of the data we added dummy codes for each country, with the USA as a reference category. We additionally calculated model 1b while grouping the data by country in order to additionally explore country specific effects, rendering separate models for each country.

In all models, we allowed the control variables of *country, social support, trust, attachment style, brooding* and *reappraisal* to covary with both latent variables. We let *age* covary with maladaptive IER as previous studies have found associations [10]. We let brooding covary with previous mental health diagnosis as strong associations have been previously found [13]. Associations with between reappraisal and clinical diagnosis have generally been found weaker [12] which is why covariance between these variables was not warranted here. Further, we let some of the scales of IER covary, as was suggested by MPlus in the modification indices function and was warranted by conceptual considerations. Specifically, we let negative and positive humor covary because both are humor based. Further, we let positive humor and co-reappraisal covary, as well as co-distraction and physical affection, as these processes might happen simultaneously. Lastly, we let negative humor and co-reappraisal covary as a scenario might apply to both.

Calculation of X^2 for interaction models

First, we ran each interaction models with the interaction terms fixed at zero as a null model in order to then be able to compare those models with the interaction models. We used the following formula to calculate the X^2 difference test for the interaction models using log-likelihood information [76]:

L0 = log-likelihood for the null model.

L1 = log-likelihood for the alternative model.

c0 = scaling correction factor for the null model.

c1 = scaling correction factor for the alternative model.

p0 = number of parameters estimated in the null model.

p1 = number of parameters estimated in the alternative model.

We calculated the value cd = (p0*c0-p1*c1)/(p0-p1).

And from that we are able to calculate the test statistic: TRd = -2*(L0-L1)/cd.

And lastly we calculate the degrees of freedom: df = p1-p0.

With the test statistic information and degrees of freedom we are then able to calculate the p-value.

For each Model specifically this was calculated as the following:

Social support interaction model:

L0 = -30704.587   L1 = -30700.798   c0 = 1.0364 c1 = 1.0398 p0 = 192 p1 = 200

cd = (192 * 1.0364 - 200 * 1.0398)/(192-200) = 1.1214

TRd = -2*(-30704.587 - [-30700.798])/1.1214 = 6.757624

df = p1-p0 = 200 -192 = 8

p-value= .56 --> **non significant**

Loneliness interaction model:

L0 = -29055.466   L1 = -30666.239   c0 = 1.0357   c1 = 1.0527 p0 = 192 p1 = 200

cd = 192 * 1.0357 - 200 * 1.0527)/(192-200) = 1.4439

TRd = -2*(-29055.466 - [-30666.239])/1.4607 = -2205.481

df = 200 -192 = 8

p-value= < .00001 --> **significant**

Attachment style interaction model:

L0 = -30747.832   L1 = -30694.841   c0 = 1.0375 c1 = 1.0387 p0 = 192 p1 = 200

cd = (192 * 1.0375 - 200 * 1.0387)/(192-200) = 1.0675

TRd = -2*(-30747.832 - [-30694.841])/1.0675 = 99.28056

df = 200 -192 = 8

p-value= < .00001 --> **significant**

Trust interaction model:

L0 = -30704.587   L1 = -30698.060   c0 = 1.0364 c1 = 1.0390 p0 = 192 p1 = 200

cd = (192 * 1.0364 - 200 * 1.0390)/(192-200) = 1.1014

TRd = -2*(-30704.587 - [-30698.060])/1.1014 = 11.85219

df = 200 -192 = 8

pvalue=   .16 --> **non significant**

SC: Tables

Table S1

IER-CR Items

Subscale	Item
	**The following questions are about dealing with emotional states. Many turn to people who are close to them to be able to cope with their emotions. Such close people could be a partner for instance**
	**When I am in a bad mood, or something is burdening me…**
Co-Distraction	.. I like to be with someone close to me because it helps me come up with other thoughts
Co-Suppression	.. I act like nothing is going on because people close to me can't help me improve my mood
Co-Brooding	.. I circle around the same topic in conversation with a person close to me, when we talk about the trigger of my mood i often do not feel understood
Co-Distraction	.. I talk to a person close to me about anything, to distract myself
Physical Affection	.. I lean on a person close to me, because it feels good to be taken into someone's arms or otherwise touched lovingly
Co-Brooding	.. I keep telling the person close to me the same thing that is burdening me, even though I know it won't change anything
Co-Suppression	.. I try to hide it from the person close to me so that they are not burdened with my bad mood
Co-Brooding	.. I often catch myself always complaining about the same things
Physical Affection	.. I look for physical comfort from a person close to me
	**When I am in a bad mood or something is burdening me, I talk to a person close to me...**
Co-Reappraisal	… so that we can together get a new perspective of things
Positive Humor	.. So that they help me improve my mood through humor
Positive Humor	.. And joke about it, so we can take things humorously together
Negative Humor	.. In order to vent with sarcastic comments
Co-Reappraisal	.. So that their perspective helps me see things in a different light
Negative Humor	.. And am bitterly ironic, because it makes things easier

Table S2
Results of model 1a & b for control variables

	**Model 1a**				**Model 1b**			
	Depressive symptoms	Adjustment disorder symptoms	Fear of COVID-19	Self-reference	Depressive symptoms	Adjustment disorder symptoms	Fear of COVID-19	Self-reference
Gender: Identified as women	.03 [-.01; .07]	.06** [.02; .11]	.03 [-.01; .08]	.17*** [.12; .22]	.03 [-.01; .06]	.06* [.01; .10]	.02 [-.02; .07]	.17*** [.12; .22]
Age	-.01 [-.07; .05]	.12*** [.07; .18]	.19*** [.13; .25]	-.08** [-.14; -.02]	-.07*** [-.11; -.03]	.08*** [.03; .13]	.15*** [.10; .20]	-.08** [-.14; -.03]
No previous mental health diagnosis	-.15*** [-.21; -.09]	-.02 [-.08; .03]	-.04 [-.10; .02]	-.08** [-.13; -.02]	-.13*** [-.18; -.09]	-.01 [-.06; .04]	-.03 [-.08; .03]	-.07** [-.13; -.02]
UK	-.06 [-.13; .00]	-.05 [-.12; .02]	-.12*** [-.19; -.05]	.04 [-.02; .10]	-.03 [-.07; .02]	-.02 [-.07; .04]	-.09** [-.15; -.03]	.04 [-.02; .10]
Germany	-.04 [-.11; .02]	.09** [.02; .16]	.07 [-.01; .14]	-.09** [-.16; -.02]	-.10*** [-.15; -.05]	.03 [-.03; .10]	.01 [-.06; .07]	-.09** [-.16; -.02]
Switzerland	-.08** [-.15; -.02]	.06 [-.01; .13]	-.01 [-.08; .07]	-.12*** [-.20; -.05]	-.10*** [-.15; -.05]	.03 [-.04; .09]	-.04 [-.10; .02]	-.11** [-.19; -.04]

Note. Significance codes *** $p < .001$; ** $p < .01$; * $p < .05$, † $p > .05$ and 95% CI which do not contain 0. 95% CI in [].The country variables were added as dummy codes with the USA as a reference category.

Table S3

Results of model 2a – d for control variables

**Model 2a: Interaction Social Support**	Depressive symptoms	Adjustment disorder symptoms	Fear of COVID-19	Self-reference	**Model 2b: Interaction Attachment**	Depressive symptoms	Adjustment disorder symptoms	Fear of COVID-19	Self-reference
Gender: Identified as women	.03 [-.01; .07]	.06** [.01; .10]	.02 [-.02; .07]	.17*** [.12; .22]		.02 [-.01; .06]	.05* [.01; .10]	.02 [-.02; .07]	.17*** [.12; .22]
Age	-.07*** [-.11; -.03]	.08** [.03; .13]	.15*** [.10; .20]	-.08** [-.14; -.02]		-.07** [-.11; -.03]	.08** [.03; .13]	.15*** [.10; .20]	-.08** [-.14; -.03]
No previous mental health diagnosis	-.13*** [-.18; -.07]	-.01 [-.06; .04]	-.03 [-.08; .03]	-.07** [-.13; -.02]		-.13*** [-.18; -.09]	-.01 [-.06; .04]	-.02 [-.08; .03]	-.08** [-.13; -.02]
UK	-.03 [-.08; .02]	-.02 [-.07; .04]	-.09** [-.15; -.30]	.04 [-.02; .10]		-.03 [-.08; .02]	-.02 [-.07; .04]	-.09** [-.15; -.03]	.04 [-.02; .10]
Germany	-.09*** [-.15; -.04]	.04 [-.03; .10]	.01 [-.06; .08]	-.09* [-.16; -.02]		-.10*** [-.15; -.04]	.04 [-.03; .10]	.01 [-.06; .08]	-.09* [-.16; -.02]
Switzerland	-.10*** [-.15; -.05]	.03 [-.04; .09]	-.04 [.10; .02]	-.11** [-.19; -.04]		-.10*** [-.15; -.04]	.03 [-.04; .09]	-.04 [-.10; .02]	-.11** [-.19; -.04]
**Model 2c: Interaction Loneliness**	Depressive symptoms	Adjustment disorder symptoms	Fear of COVID-19	Self-reference	**Model 2d: Interaction Trust**	Depressive symptoms	Adjustment disorder symptoms	Fear of COVID-19	Self-reference
Gender: Female	.03 [-.01; .07]	.05* [.01; .10]	.02 [-.02; .07]	.18*** [.13; .23]		.02 [-.02; .06]	.06* [.01; .10]	.02 [-.02; .07]	.17*** [.12; .22]
Age	-.07** [-.11; -.02]	.09*** [.04; .14]	.16*** [.11; .21]	-.09** [-.14; -.03]		-.07** [-.11; -.02]	.08** [.03; .13]	.15*** [.10; .20]	-.08** [-.14; -.02]
No previous mental health diagnosis	-.13*** [-.18; -.08]	-.01 [-.06; .04]	-.03 [-.08; .02]	-.08** [-.13; -.02]		-.13*** [-.18; -.09]	-.01 [-.06; .04]	-.03 [-.08; .02]	-.07** [-.13; -.02]
UK	-.03 [-.08; .02]	-.02 [-.08; .03]	-.09** [-.15; -.03]	.05 [-.01; .10]		-.03 [-.08; .02]	-.02 [-.08; .04]	-.09** [-.15; -.03]	.04 [-.02; .10]
Germany	-.09** [-.14; -.03]	.04 [-.03; .11]	.01 [-.06; .08]	-.09* [-.16; -.02]		-.09*** [-.15; -.04]	.04 [-.03; .10]	.01 [-.06; .08]	-.09* [-.16; -.02]
Switzerland	-.09*** [-.15; -.04]	.03 [-.04; .09]	-.04 [-.11; .02]	-.12** [-.19; -.04]		-.10*** [-.15; -.05]	.03 [-.03; .09]	-.04 [-.10; .03]	-.12** [-.19; -.04]

Note. Significance codes *** $p < .001$; ** $p < .01$; * $p < .05$, † $p > .05$ and 95% CI which do not contain 0. 95% CI in [].The country variables were added as dummy codes with the USA as a reference category.

Table S4
Results of structural model for USA

	Depressive symptoms	Adjustment disorder symptoms	Fear of COVID-19	Self-reference
Adaptive IER	-.10 [-.23;.04]	-.06 [-.22;.10]	-.06 [-.23;.12]	.15 [-.04;.33]
Maladaptive IER	.21** [.05;.36]	.22* [.04;.41]	.21* [.01;.41]	-.31** [-.52;-.09]
Brooding	.38*** [.28;.48]	.37*** [.24;.49]	.33*** [.20;.46]	.20** [.06;.35]
Reappraisal	-.13** [-.20;-.05]	.05 [-.04;.14]	.05 [-.05;.14]	.00 [-.10;.10]
Social Support	-.02 [-.11;.06]	.04 [-.06;.15]	-.02 [-.14;.09]	-.09 [-.21;.03]
Attachment	-.01 [-.09;.07]	.01 [-.09;.11]	.03 [-.08;.13]	-.05 [-.17;.06]
Loneliness	.22*** [.13;.31]	.18*** [.07;.29]	.11 [-.01;.23]	.10 [-.02;.22]
Trust	-.01 [-.08;.07]	-.01 [-.10;.08]	-.00 [-.10;.10]	-.06 [-.16;.04]

Note. All dependent variables were further controlled for sex, age, previous mental health diagnosis. Significance codes *** $p < .001$; ** $p < .01$; * $p < .05$, † $p > .05$ and 95% CI which do not contain 0. 95% CI in []. IER stands for interpersonal emotion regulation.

Table S5
Results of structural model for UK

	Depressive symptoms	Adjustment disorder symptoms	Fear of COVID-19	Self-reference
Adaptive IER	.03 [-.16;.22]	.06 [-.17;.29]	.27* [.05;.49]	-.04 [-.28;.20]
Maladaptive IER	.11 [-.08;.30]	.28* [.05;.52]	.04 [-.20;.27]	-.03 [-.28;.22]
Brooding	.29*** [.19;.39]	.24*** [.12;.36]	.30*** [.18;.42]	-.03 [-.16;.10]
Reappraisal	-.12** [-.20;-.04]	-.01 [-.10;.09]	-.05 [-.15;.05]	-.08 [-.19;.03]
Social Support	-.02 [-.12;.08]	-.01 [-.12;.10]	-.07 [-.18;.06]	.03 [-.10;.16]
Attachment	-.02 [-.11;.06]	-.02 [-.12;.08]	.01 [-.10;.12]	.05 [-.06;.17]
Loneliness	.20*** [.10;.30]	.12 [-.00;.24]	.11 [-.02;.23]	.16** [.03;.29]
Trust	-.06 [-.14;.02]	-.01 [-.10;.08]	-.09 [-.19;.00]	-.03 [-.13;.07]

Note. All dependent variables were further controlled for sex, age, previous mental health diagnosis. Significance codes *** $p < .001$; ** $p < .01$; * $p < .05$, † $p > .05$ and 95% CI which do not contain 0. 95% CI in []. IER stands for interpersonal emotion regulation.

Table S6
Results of structural model for Germany

	Depressive symptoms	Adjustment disorder symptoms	Fear of COVID-19	Self-reference
Adaptive IER	-.03<br>[-.23;.17]	.18<br>[-.04;.40]	.11<br>[-.13;.36]	.17<br>[-.11;.44]
Maladaptive IER	.23<br>[-.04;.50]	.16<br>[-.13;.45]	.36*<br>[.04;.68]	-.29<br>[-.65;.07]
Brooding	.20*<br>[.04;.36]	.20*<br>[.02;.38]	.17<br>[-.02;.36]	.07<br>[-.15;.29]
Reappraisal	-.07<br>[-.18;.04]	-.06<br>[-.19;.06]	.05<br>[-.08;.19]	-.11<br>[-.25;.05]
Social Support	-.05<br>[-.15;.05]	-.12*<br>[-.23;-.01]	-.14*<br>[-.26;-.02]	.04<br>[-.09;.17]
Attachment	-.08<br>[-.19;.03]	.00<br>[-.12;.13]	.00<br>[-.13;.13]	.03<br>[-.12;.18]
Loneliness	.32***<br>[.22;.43]	.25***<br>[.12;.37]	.00<br>[-.13;.13]	.16*<br>[.01;.30]
Trust	.02<br>[-.07;.11]	-.01<br>[-.11;.09]	.02<br>[-.08;.12]	.02<br>[-.10;.13]

Note. All dependent variables were further controlled for sex, age, previous mental health diagnosis. Significance codes *** $p < .001$; ** $p < .01$; * $p < .05$, † $p > .05$ and 95% CI which do not contain 0. 95% CI in []. IER stands for interpersonal emotion regulation.

Table S7
Results of structural model for Switzerland

	Depressive symptoms	Adjustment disorder symptoms	Fear of COVID-19	Self-reference
Adaptive IER	-.15<br>[-.31;.02]	.12<br>[-.06;.30]	-.01<br>[-.20;.17]	.15<br>[-.06;.36]
Maladaptive IER	.16<br>[-.04;.36]	.32**<br>[.10;.55]	.32**<br>[.08;.55]	-.14<br>[-.40;.12]
Brooding	.24***<br>[.10;.39]	.12<br>[-.05;.28]	.18*<br>[.01;.35]	.00<br>[-.19;.19]
Reappraisal	-.06<br>[-.16;.04]	-.13*<br>[-.24;-.02]	-.09<br>[-.21;.02]	-.08<br>[-.21;.05]
Social Support	.04<br>[-.07;.15]	.07<br>[-.05;.20]	.15*<br>[.02;.28]	-.08<br>[-.23;.07]
Attachment	.01<br>[-.10;.11]	.03<br>[-.09;.15]	.01<br>[-.11;.13]	-.12<br>[-.25;.02]
Loneliness	.37***<br>[.27;.47]	.25***<br>[.13;.36]	.14*<br>[.02;.26]	-.03<br>[-.16;.10]
Trust	-.13**<br>[-.22;-.04]	-.09<br>[-.19;.01]	-.10*<br>[-.20;-.00]	.01<br>[-.10;.13]

Note. All dependent variables were further controlled for sex, age, previous mental health diagnosis. Significance codes *** $p < .001$; ** $p < .01$; * $p < .05$, † $p > .05$ and 95% CI which do not contain 0. 95% CI in []. IER stands for interpersonal emotion regulation.
